# Peritoneal Catheters Malposition/Dysfunction and Their Approach with Catheterography and Radiologic Manipulation in Peritoneal Dialysis: A Minireview and Case Series

**DOI:** 10.3390/life14111475

**Published:** 2024-11-13

**Authors:** Martina Cacciapuoti, Anna Basso, Lucia Federica Stefanelli, Federico Nalesso, Lorenzo A. Calò

**Affiliations:** Nephrology, Dialysis and Transplantation Unit, Department of Medicine, University of Padova, 35128 Padova, Italy; martina.cacciapuoti@phd.unipd.it (M.C.); anna.basso@aopd.veneto.it (A.B.); luciafederica.stefanelli@unipd.it (L.F.S.); federico.nalesso@unipd.it (F.N.)

**Keywords:** peritoneal dialysis, catheter malfunction, catheterography, radiologic manipulations, peritoneal catheter

## Abstract

Peritoneal catheter dysfunction is one of the most frequent complications of peritoneal dialysis. The malposition of a peritoneal catheter may cause one- or two-way obstruction with fluid outflow or inflow problems, large residual volumes, and, therefore, reduced ultrafiltration and sometimes abdominal pain. Standard procedures may often fail to solve the dysfunction. Catheterography is an interventional radiologic procedure based on the infusion under aseptic conditions of iodated contrast into the peritoneal catheter, followed by the introduction of a guidewire into the catheter for guidewire manipulation. The available literature about catheterography is quite scarce and mainly based on case reports, case series, and small retrospective studies. In this minireview, we describe the guidewire manipulation techniques explored so far and their pros and cons. In addition, four interesting cases of catheterography performed in our center are also reported. In conclusion, in this minireview, the pros and cons of catheterography have been outpointed. Radiologic manipulation of peritoneal catheters may represent an effective and safe solution for malfunctioning peritoneal catheters and may also be exploited as “bridge therapy” to laparotomy in patients temporarily unsuitable for surgery. The advantages of this procedure are that it does not require long-term hospitalization and allows immediate resume of peritoneal dialysis.

## 1. Introduction

Peritoneal catheter dysfunction is one of the most frequent complications of peritoneal dialysis [1], with a reported incidence of 5.5–55% [1]. As recommended in the International Society of Peritoneal Dialysis Guidelines, the tip should be located in the pelvis for optimal hydraulic function. The malposition of a peritoneal catheter may cause one-way or two-way obstruction with fluid outflow problems—and, less frequently, inflow—and sometimes abdominal pain. The catheter dysfunction may also manifest as drain alarms in Automated Peritoneal Dialysis (APD), large residual volumes, and, therefore, reduced ultrafiltration and impaired clearance [1].

Two-way obstruction is usually secondary to fibrin plugs. One-way obstruction may be attributed to constipation and dilated sigmoid colon, catheter displacement out of the pelvis, entrapment in the omentum or bowel, or compartmentalization by adhesions. Much more rare is the obstruction due to extreme bladder distension secondary to bladder outlet obstruction [1].

Loculations or adhesions around the catheter tip are inevitable consequences of their intraperitoneal position. Patients with a history of peritonitis are more prone to develop adhesions that often form a tunnel around the catheter tip, letting the fluid pass in but not out, as the adhesions are sucked against the catheter during the outflow phase. Filling defects within the distal end of the Tenckhoff catheter at the catheterography may be a sign of adhesions attached to the catheter and insinuated within the side holes [2].

The incidence of catheter outflow failure is reported to be higher after open surgical and blind guidewire/trocar catheter placement (10–22%) than after laparoscopic intervention (4–13%) [3].

The Society of American Gastrointestinal and Endoscopic Surgeons (SAGES) 2023 Guideline Update suggests that catheter dysfunction should be managed with a logical ascendent order from the least to the most invasive procedure. Therefore, the first suggestion for the patient with a malfunctioning catheter would be an adequate stool evacuation, as a distended rectosigmoid colon may obstruct the side catheter holes or set the catheter tip in a poor functioning position. The aspect of the effluent fluid is also to be taken into account, as if it contains fibrin strands, the catheter dysfunction may be due to fibrin clots that may easily be cleared with the infusion of thrombolytic solutions. If the catheter dysfunction perpetuates, an abdomen X-ray can easily show tubing kinks or displacement. However, kinks mostly occur in the transmural segment of the catheter and are, therefore, hard to demonstrate on flat-plate radiographs. Lateral films of the abdomen, while the patient is supine and sitting, may help detect kinks. Obstructions by adhesions may be hard to recognize with radiological imaging as the position may appear normal. Further management strategies are catheterography or laparoscopy with catheter repositioning, adhesiolysis, omentectomy, or omentopexy if nonoperative techniques fail to solve the problem [1].

Catheterography is an interventional radiologic procedure based on the infusion under aseptic conditions of iodated contrast (usually 10–20 mL) into the peritoneal catheter. During the contrast infusion, serial images of the catheter are recorded. Further introduction of a guidewire into the catheter for guidewire manipulation is then possible. Yang et al. reported the employment of CT instead of plain Rx [4].

An advantage of this technique is the low invasivity and discomfort for the patient and the possibility of using the catheter right after the procedure, if successful. However, the rate of success is not high, ranging between 46 and 75%, and sometimes it is even impossible to determine the underlying cause of the flow dysfunction. The extraperitoneal location of the peritoneal catheter is an obvious contraindication to manipulation [5], and a trained and expert radiologist is required. These drawbacks inevitably affect patients’ perception of the problem, and the interruption of the dialytic therapy may dissuade them from continuing with peritoneal dialysis.

In the SAGES 2023 Guideline Update it is reported that nonoperative rescue benefits of a lower rate of bleeding, exit site infection, and peritonitis compared to operative intervention (respectively, 1.1% vs. 3.3%; 0.94% vs. 6.6%; 1.1% vs. 7.1%) but is burdened by a higher risk of early and late catheter dysfunction (36.9% vs. 18.55; 62.4% vs. 25.6%, respectively) [1].

Exploratory laparoscopic surgery is considered the definitive care by the SAGE 2023 Guideline Update; therefore, it is to be taken into account especially if the patient is in urgent need of dialysis [1]. If this approach is not feasible, catheter replacement is required [6].

The available literature about catheterography is quite scarce and mainly based on case reports, case series, and small retrospective studies.

In this minireview, we describe the guidewire manipulation techniques explored so far and factors related to their effectiveness. Four interesting cases of catheterography performed in our center are also reported.

We performed a non-systematic minireview of the literature using the PubMed database from inception to 10 February 2024, using the search terms “catheterography in peritoneal dialysis”, “peritoneal catheter radiological manipulation”, and “fluoroscopic manipulation of peritoneal catheter”. Only original articles (retrospective studies, case series, and case reports) written in English with full-text links were included. Papers dealing with catheters other than peritoneal catheters (e.g., feeding tubes) were obviously excluded. Papers dealing with the manipulation of catheters placed by interventional radiologists or implying mini-surgical techniques were excluded. The results of the literature search are reported in Table 1.

## 2. Techniques

Authors reported different techniques with the use of a variety of guidewires. The most adopted technique is the so-called “double-guidewire” technique. Very similar to that of Jacques et al. [7], in 1994, Siegel et al. introduced the double guidewire technique by inserting the stiffener through a biliary drainage catheter over the guidewire after the repositioning of the catheter into the pelvis. The stiffener was used to anchor the catheter in the new position and allow safe removal of the guidewire [5].

In 1996, Lim et al. described a technique of electrocauterization—first performed in vitro—to recanalize catheters with ingrowing omental fat. The diagnosis of the presence of ingrowing omental fat was evident if no spillage of contrast medium could be visualized either at the side holes or at the tip end. A stone basket was used as a conductor of electricity and to remove the fragments of cauterized ingrowing omental fat, as performed in the in vitro experiments. Endoluminal cauterization was performed by inserting the stone basket into the Tenckhoff catheter under fluoroscopic guidance until the obstructed distal tip. Cauterized omental fat was then removed with the stone basket. In case of catheter malposition, a stiff guidewire would be employed to relocate it in the rectovesical pouch, if necessary, with a supplementary help of a metal stylet used to free the catheter tip from loculations and adhesions. The rate of effectiveness was 4 over 6, and no severe complications (such as omental hemorrhage or bowel perforation) occurred [2].

Lee et al. adopted the same concept in 2003 to overcome two issues encountered with the single-wire procedure: the first is the back migration of the catheter after the removal of the guidewire; the second is the patient abdominal discomfort and potential for bowel injury triggered by the stiffness of the Lunderquist guidewire. Their double guidewire technique consisted of the employment of a flexible curved-tip, fixed-core guidewire with a polytetrafluoroethylene (PTFE) coating that was inserted through the catheter to buckle it and direct the catheter tip downward to the pelvic cavity. When this procedure was not effective, a second guidewire was pushed within the catheter lumen to prevent backward flipping of the catheter by withdrawing the first guidewire. In the end, the second guidewire was also gently removed. The success of the rescue was ascertained without the need for contrast agents just by evaluating the duration time of infusion and draining of the peritoneal dialysis solution: if they were 8 to 10 min and 20 to 30 min, respectively, the intervention was considered successful. The immediate success of this technique was quite high (86%), and no adverse event was reported. There are four distinct advantages of this technique. First, the PTFE-coated guidewire is of moderate flexibility and softness and thus is less likely to result in abdominal pain or discomfort, as noted during the procedure. Second, the curved-tip design decreases the likelihood of bowel injury. Third, compared to the stiff Lunderquist guidewire, PTFE-coated guidewires are easier to manipulate, and the first guidewire can easily be buckled back into the pelvic cavity so that the migrated catheter can be redirected downward. Fourth, the second guidewire can also be inserted with ease to anchor the catheter and can be removed without backward flipping of the catheter so that remigration can be prevented. The whole procedure can be accomplished in approximately 5 min [8].

Ozyer et al. and Degesys et al. adopted a very similar technique based on the use of a rod that was inserted into the peritoneal catheter over the guidewire to facilitate both the repositioning of the catheter tip and the tearing of adhesions [9,10]. Ozyer et al. declared they did not need contrast agent as the catheters were easily seen fluoroscopically. Thanks to the angular shape of the rod, it was possible to tear apart adhesions and fibrotic bands, reducing the chance of remigration. This technique was proposed after acknowledging that the repositioning with the bare guidewire was poorly efficient. Ozyer reported a durable success (>1 month) in 9 out of 11 procedures and the need for remanipulations in only two cases, both successful. In the report of Degesys et al., the durable success rate was 58%, while Siegel et al. reported the lowest long-term effectiveness of a similar technique (42%). An important complication that occurred in the cohort of Ozyer et al. was pain, reported despite iv sedation with midazolam and fentanyl and peri-exit site local anesthetic pre-treatment with prilocaine [10].

In 2012, Miller et al. described the use of a guidewire to clear fibrin clots into the catheter and to straighten the coiled tip. To achieve the repositioning in case of migration, the guidewire was angulated before insertion into the catheter [11].

In 2015, Saka et al. presented a procedure with the utilization of an “alpha-replacer”, which is a special guidewire that becomes flexible if straightened and harder when coiled. This unique feature appeared to be especially effective in the repositioning of catheters [12]. Asai et al. explored the utilization of the same guidewire for obstructed catheters and found a high success rate (87.5%). However, they reported they did not try thrombolytic infusion and went straight to the radiologic manipulation [13].

In 2021, Li et al. performed two different techniques based on the etiology of catheter malfunctioning. In case of malposition, a metal stiffener was inserted approximately two-thirds into the catheter using an 8.5-F biliary drainage catheter set (Cook Medical, Bloomington, Indiana) to relocate the catheter into the pelvis. If the catheter was obstructed, two 145 cm, 0.035-inch hydrophilic guidewires (Terumo, Somerset, NJ, USA) with a 3 cm flexible tip length were advanced so that the tips of the wires were positioned 1 cm beyond the tip of the PD catheter. The guidewires were rapidly rotated using Kelly hemostatic forceps for 15–30 rotations and removed. Contrast medium was then infused, and if the catheter was not patent yet, two 145 cm, 0.035-inch Amplatz wires (Cook Medical, Bloomington, Indiana) were inserted so that the tips of the wires were 1 cm beyond the tip of the catheter and likewise rotated and then removed. In this case, the key modification was the utilization of rotating guidewires for catheter recanalization and a metallic stiffener for repositioning. This technique was particularly efficient in cases of malpositioned catheters, with a clinical success after 30 days of 77%. Obstructed catheters did not benefit as much from this technique, with a success rate of only 43% [6].

## 3. Effectiveness

The effectiveness of the manipulation was differently categorized. Most of the authors distinguished “immediate success”, i.e., patency of the catheter right after the procedure, from “durable success”, which was usually defined as the functioning of the catheter one month—or 30 days—after the manipulation. Some authors also evaluated “1 week success” and functioning of the catheter after 3, 6, and 12 months, which was only reported by Savader et al. [14]. Dobrashian et al. differentiated “technical success”, i.e., the successful repositioning at screening, from “clinical success”, i.e., continued effective Continuous Ambulatory Peritoneal Dialysis (CAPD) 6 months thereafter [15] (Table 2).

The immediate success rate in the evaluated studies was 78–93%, while the durable success rate was 25–82%.

The authors speculated that some factors may influence the success of the manipulation: time from placement, location of the catheter, previous surgery, or peritonitis.

According to Savader et al., durable patency (>30 days) was more likely to be achieved after radiologic manipulation if the malfunction occurred more than 1 month after placement compared to less than one month after placement (65% vs. 25%, *p* = 0.08). This was also true for secondary manipulation. The proposed explanation for this is that early failure may be attributed to poor initial catheter positioning, a problem that may not be easily corrected by radiologic manipulation. Late failures instead may be attributable to a fibrinous peri-catheter sheath or catheter entrapment by adhesions, a problem that can be managed by guidewire manipulations that might disrupt and free up the catheter [14]. At odds with these observations, however, Li et al. found a higher success rate (both immediate and durable) of manipulations of malpositioned catheters (77%) than of obstructed ones (43%) [6].

Degesys et al. observed that the initial and durable success rates after manipulation were higher after a longer time of proper catheter function [10], and Saka et al. came to the same conclusion [12]. This observation has led to the suggestion that although every patient should first be treated with radiological manipulation, a second attempt in case of early catheter dysfunction is probably helpless, and a surgical solution should be sought [10]. This aspect, however, was not confirmed by the same group, who later found no correlation between the length of functional catheter life and manipulation success rate [16]. Li et al. also came to the same conclusion [6].

Miller et al. also reported 70 cases of radiologic manipulation of previously embedded catheters, distinguishing “primary failure”—dysfunction right after the exteriorization of the catheter—from “secondary failure”—malfunctioning after a period of proper functioning. Their analysis showed that the success of the procedure was higher in case of secondary failure than primary failure and that catheters placed in the pelvis—instead of the upper abdomen—were more likely to function after radiological manipulation (73.5% vs. 42.9%, *p* = 0.01) [11].

At odds with these findings, Siegel et al. demonstrated that early catheter failure was more amenable to catheter manipulation vs. late catheter failure (75% vs. 44%) and made suppositions that this was attributable to fibrin sheath formation. In fact, fibrin sheaths take time to develop, and, therefore, catheter malfunction secondary to other causes may benefit more from radiological manipulations [6]. In support of this, Dobrashian et al. suggested that longer peritoneal dialysis catheter duration (>1 year), previous history of surgery, and peritonitis were risk factors for technical failures, and this was attributed to the higher likelihood of adhesions formation [15]. Ozyer et al. attributed their high success rate (93%) to the fact that patients who underwent the radiological manipulation procedure did not have a history of peritonitis or abdominal surgery [9]. Moreover, Dobrashian et al. noticed that there was a trend toward a higher failure rate with increasing bodyweight, although without statistical significance [15].

Small sample populations may explain this discrepancy between studies.

Regarding the catheter position, Dobrashian et al. analyzed the position of the catheter before manipulation and noticed three different patterns of migration, and each was treated with different techniques. The position with the catheter tip pointing laterally against the abdomen–pelvic side wall was the one with higher success rates compared to the catheter tip pointing toward the hypochondrial region or with the catheter looped back on itself [15].

In Miller et al., the cases in which radiologic manipulation failed were addressed to laparoscopic intervention (18/26) that revealed the causes of mechanical malfunction: omentum and adhesions or loops of bowel entrapment and obstruction by fibrin or blood. In two cases, the cause was not detected [11].

The arcuated shape of the swan-neck peritoneal catheter was considered an obstacle to the radiological intervention; however, in the cohort of Saka et al., the effectiveness rate of the manipulation of swan-neck catheters was close to the other procedures [12].

Moreover, Moss et al. observed that patients receiving PD for acute renal failure were more likely to attain a benefit from stiff wire manipulation. None of the other evaluated studies on the topic reported cases of patients with PD for acute renal failure [16].

Different authors reported that some patients required multiple manipulations after the failure of the first attempt or recurrence of migration. Lee et al. mentioned that two patients in whom there had been immediate success with the procedure underwent a second manipulation and eventually had their catheters removed due to repeated migrations [8]. Dobrashian et al. reported that up to one-third of the patients whose procedures were considered immediately successful experienced remigration of the catheter within 6 months, and most of these patients underwent another manipulation, and one patient, even a third one [15].

Siegel et al. demonstrated that multiple attempts to reposition malfunctioning peritoneal catheters were worthwhile: 36% (five of 14 catheters) with durable success were manipulated more than once, and of those manipulated more than once, 50% (five of 10 catheters) had durable success. Therefore, this author suggests that radiological manipulations may be attempted up to four times before addressing patients to surgical removal, replacement, or switch to a different modality of RRT [5].

Savader et al. also reported a high number of remanipulations, and a further increase in the rate of durable success was achieved [14].

The “shape memory”—the tendency of the catheter to return to a stretched position—may influence the rate of dislocation. In fact, patients who had their catheters replaced by another single-cuff Tenckhoff catheter were likely to continue to have problems with malposition. Moss et al. suggested that the factors that led to the initial malposition (such as body habitus, extremes of size, abdominal adhesions, or large omentum) remain present, perpetrating problems of malposition [16].

Therefore, available data do not allow us to draw a conclusion about this aspect: some authors would suggest repeating multiple manipulations, and others would switch to surgical replacement right after the first radiologically guided manipulation’s failure.

## 4. Complications

The main complications of peritoneal catheter guidewire manipulation are peritonitis and abdominal pain.

Antibiotic prophylaxis is usually administered before the procedure, but some authors reported administration after the maneuver [17] or even none [12]. Peritonitis incidence was averagely 4.8% in the studies included in this minireview. Kim et al. observed a lower incidence of peritonitis during the follow-up period in the fluoroscopically guided wire manipulation in comparison with the laparoscopic surgery, but the time lag between the procedures and the peritonitis episode was averagely of 7 months and no episode was attributed to the procedures [18]. A consensus about the lag of time between the procedure and the occurrence of peritonitis to attribute the infectious event to the maneuver is yet to be reached. Abdominal pain may occur despite sedation [9]. The administration of contrast medium did not lead to any significant difference in urine volume output [13]. Very rare complications are abdominal wall leak [19] and the rupture of the balloon catheter within the Tenckhoff catheter, a condition that required laparotomy for catheter replacement and removal of the fragment [15].

## 5. Future Perspectives

As suggested by Miller et al., further opportunities for research include determining if there are other variables that are important for successful fluoroscopic manipulation, such as the appearance of the abdomen at the time of catheter insertion, the insertion technique, and the expertise of the radiologist. A randomized controlled trial comparing the results of fluoroscopic versus laparoscopic manipulation of failed peritoneal catheters would eventually help to provide conclusive evidence with respect to success rates, cost, and patient acceptance of the two procedures [13].

Targeted algorithms based on malfunction etiology—which is not always easy to diagnose—are also warranted.

Finally, it is desirable that every center refers to the same definitions of patency duration and time between procedure and complications occurrence in order to be able to improve follow-up of the patients in different cohorts and to allow comparisons among them.

## 6. Case Series

In the Padova University Hospital, Padova, Italy, in case of peritoneal catheter malfunction, we usually proceed with laxatives administration and with the instillation of thrombolytics, especially in case of the presence of fibrin strands in the effluent solution. If these strategies fail to solve the problem, an abdomen X-ray usually helps to diagnose the reason for malfunction and to guide further treatment. Every patient is usually first addressed to radiological guidewire manipulation, and in case of failure, surgery consultancy is then indicated for possible laparoscopic revision of the catheter. Second manipulations have never been recorded in our center.

In our center, catheterography with radiological guidewire manipulation is usually performed by two expert interventional radiologists in outpatient regimen. The guidewire utilized is the Flexima “APD”. Antibiotic prophylaxis with cefazolin—or vancomycin in the case of an allergy to penicillins—is usually administered before the intervention. A dialysis nurse assists with the procedure and checks the catheter patency with glucose-based solution infusion at the end of the procedure. The patient is then warned to report any adverse event, such as fever, abdominal pain, exit site signs of infection, or inflow or outflow difficulties. The patients resume their peritoneal dialysis prescription right after the intervention. The finding of filling defects within the catheter and the outlining of villous-like material close to the catheter make it suspicious for the presence of adhesions. Also, pseudocele formation and uneven distribution of the contrast suggest omental wrapping. Also, the absence of spillage of contrast medium from side holes suggests there might be fibrin or omental tissue obstructing the holes.

### 6.1. Case 1

**Figure 1:**
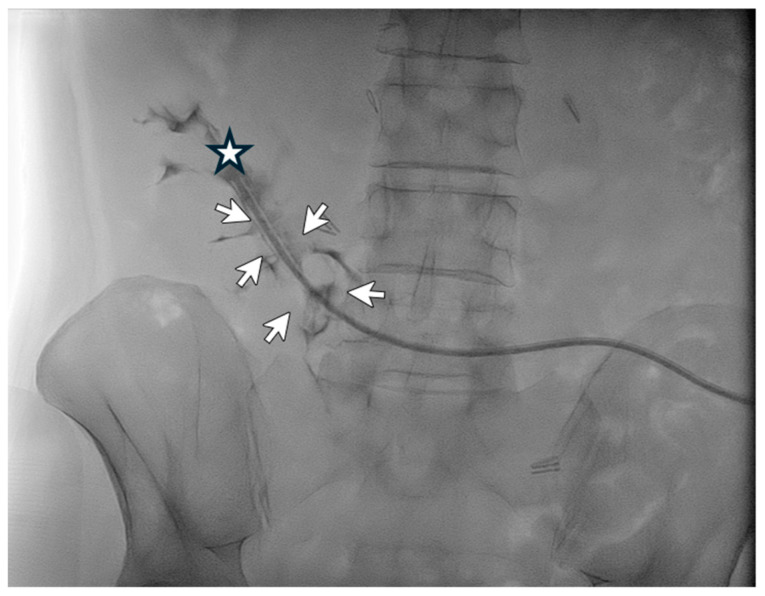
Catheterography showing the tip (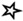) of the peritoneal catheter pointing to the lumbar right region. Normal spillage of the contrast medium from the side holes may be seen (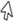).

A 48-year-old male patient affected by ADPKD underwent right nephrectomy and concurrently videolaparoscopic peritoneal catheter placement. Two months later, due to persistent abdominal bulk secondary to the contralateral kidney size, the patient underwent a second surgery for left nephrectomy. Three months after the start of peritoneal dialysis, poor inflow and outflow performance was detected with little benefit from laxatives administration. An abdomen X-ray showed the peritoneal catheter rising from the lumbar left region with the tip ending in the lumbar right region (Figure 1).

The next day—after adequate antibiotic prophylaxis—the patient underwent catheterography and radiological manipulation of the catheter with repositioning of the catheter tip in the pelvis. Afterward, the patient could immediately and effectively resume peritoneal dialysis, and no complication of the procedure occurred. The peritoneal catheter was well-functioning one month after the procedure.

### 6.2. Case 2

**Figure 2:**
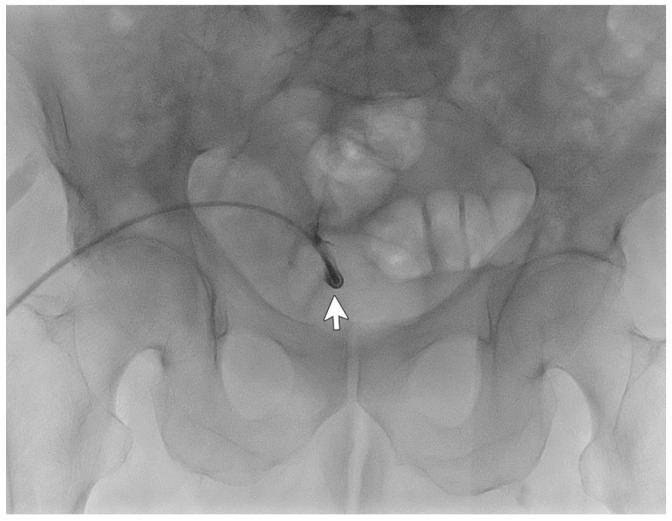
Catheterography showing the peritoneal catheter with the tip (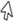) ending in the right iliac region.

A 77-year-old male patient with ESKD secondary to diabetic kidney disease underwent placement of a “Vicenza Short”peritoneal catheter by open surgical dissection under local anesthesia. Peritoneal dialysis was started after two weeks, but poor drainage performance with a large residual volume was immediately observed. An abdominal X-ray documented coprostasis and the right location of the peritoneal catheter tip in the pelvis. The patient tried different laxatives and enemas without improvement in the outflow performance of the catheter. The patient underwent catheterography that demonstrated regular inflow but impeded outflow function. The catheter appeared to be stuck on the postero-lateral side of the iliac right region (Figure 2), and wire-guided manipulations failed to move it from its original position. The patient reported mild pain after the procedure that was easily relieved with oral acetaminophen.

The CT abdomen scans disclosed adipose tissue entrapping the peritoneal catheter near its insertion in the abdominal cavity and the tip being right before the bladder wall. Considering the patient’s comorbidities, he was not eligible for videolaparoscopic rescue and thus was subjected to peritoneal catheter removal and replacement with a longer one (Vicenza) through a mini-laparotomy surgery. This catheter showed better hydraulic function and allowed the patient to finally perform adequate peritoneal dialysis.

In this case, catheterography failed to diagnose the cause of malfunctioning, and the CT scan lateral film aided it.

### 6.3. Case 3

**Figure 3:**
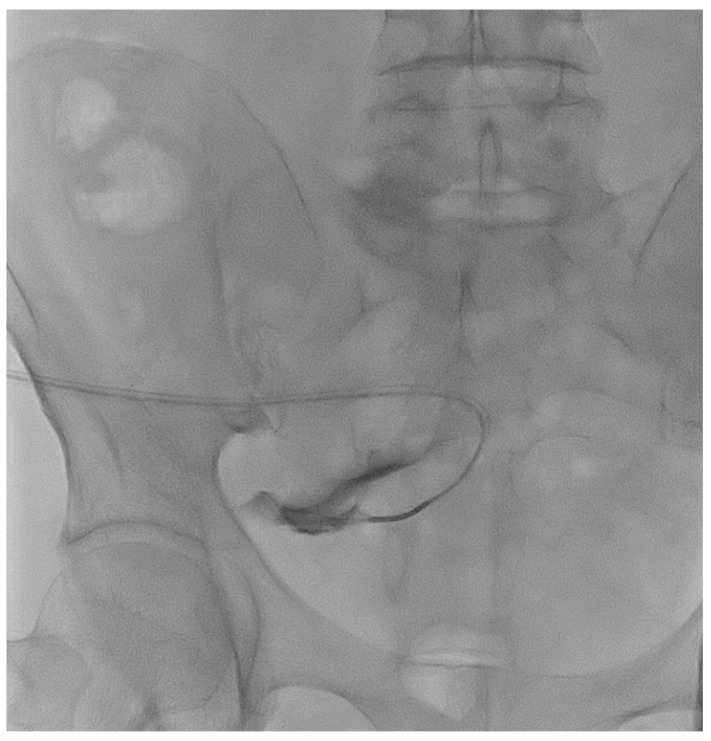
Catheterography showing no spillage of contrast medium from the side holes.

A 67-year-old male patient affected by Autosomal Dominant Tubulointerstitial Kidney Disease due to UMOD mutation (ADTKD-UMOD) in peritoneal dialysis for 15 months reported catheter malfunction since the start of peritoneal dialysis with many alarms occurring during the outflow phase of every APD cycle. The abdomen X-ray showed the correct position of the catheter tip, and laxatives did not help to reduce alarm frequency. Guidewire manipulation was successfully performed (Figure 3). The absence of spillage from the side holes made it suspicious for the presence of adhesions insinuated into the holes that may explain the drainage problems. Also, the use of the guidewire to force the movement of the catheter from its position may have helped to debride the catheter from adhesions. The patient did not report pain or any other complication of the procedure and could re-take peritoneal dialysis right afterward. One month after the procedure, the patient could still effectively perform peritoneal dialysis.

### 6.4. Case 4

**Figure 4:**
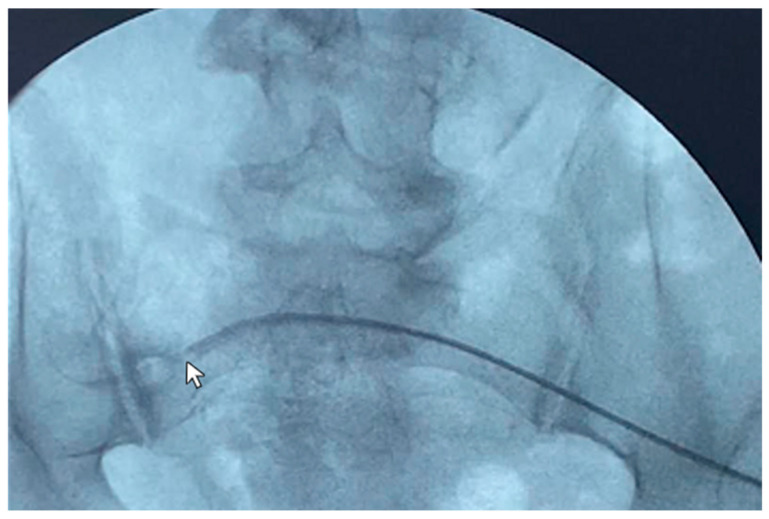
Use of the guide (Wire M—stiff type angled) to reposition the catheter tip (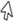)—pointing against the right lumbar region—under radioscopic guidance.

A 79-year-old female patient in peritoneal dialysis for 14 months for diabetic kidney disease was diagnosed with malfunction of the peritoneal catheter with inflow and outflow problems. The patient was thus subjected to catheterography, which showed the tip ending stuck against the right lumbar region (Figure 4). The guidewire manipulation did not manage to reposition the catheter in the pelvis, as the catheter was probably blocked by consolidated adhesions anchoring it to the abdominal side wall. No complication was reported. The patient dropped out of peritoneal dialysis and switched to hemodialysis by choice.

## 7. Conclusions

In this minireview, different peritoneal catheter guidewire manipulation techniques have been explored and compared.

We have found an average durable success rate of radiologically guided manipulation of 25–82%. A consensus about the definition of “durable success” is yet to be reached. This result arguably overestimates the real effectiveness of this technique if selection bias in the analyzed studies—severe cases would be addressed directly to laparoscopic treatment—is taken into account.

According to our literature research results, in our case series, we report an immediate success rate of 50% and a 1-month success rate of 100%. No significant complication was recorded. The influence of different factors, such as time from insertion, cause of malfunction, primary or secondary failure, previous peritonitis or surgery, etc., is hard to define, considering the observational nature of the available studies. Further studies are required to standardize techniques and create solid algorithms based on assumed causes of catheter dysfunction.

## Figures and Tables

**Table 1:** Results of literature search (criteria reported in the text).

Paper Type	Number of Papers
Retrospective observational studies	16
Case series	1
Case reports	2

**Table 2:** Characteristics and results of the considered studies.

Authors, Year	Type of Study	N of pt./ Pro.	Type of Catheter	Indications to Proc.	Type of Wire	Definition of Effectiveness	Effective Cases	Complications
Jacques et al., 1980 [7]	Retrospective study	18 pt.	Tenckhoff	2 visceral discomfort 5 early catheter blockage 11 late catheter blockage	Polyethylene catheter and guidewire; stiff-rod technique in case of failure	“satisfactory result”: relief of visceral discomfort/return to normal in- and outflow	“satisfactory result”: 77%	Pain
Degesys et al., 1985 [10]	Retrospective study	28 pt, 50 proc.	Tenckhoff catheters	5 visceral pain 8 poor inflow due to kink (3), blood clot (3), or fibrin deposit (2) 37 poor outflow	Metal rod	DS: long-term, i.e., > 1 mo. catheter function, improvement in renal function of termination of PD in case of AKI, relief of pain	IS: 88% DS: 58%	None
Moss et al., 1990 [16]	Retrospective study	48 proc.	Single-cuff catheters	Malposition	Metal rod	IS 1-week success 1 mo. success (long-term catheter salvage)	IS: 78% 1-week success: 51% Long-term catheter salvage: 25%	Pain, peritonitis (1 pt)
Siegel et al., 1994 [5]	Retrospective study	25 pt	Tecnkhoff catheters	22 outflow failure 3 painful dialysis (shoulder or pelvic pain)	Amplatz Super Stiff; Medi-tech/Boston Scientific	IS 1-week success DS	IS: 89% 1-week success: 55% DS: 43%. Repeated manipulations x2: 8 pts with DS in 38% Repeated manipulations x3: 1 pt with DS; x4: 1 pt with DS	Respiratory arrest after sedation in an obese patient (1 pt); catheter dislodgement that required surgical repositioning (1 pt)
Lim et al., 1996 [2]	Case series	7 pt	Tenckhoff catheters	Outflow difficulty	Stiff guidewire Amplatz super stiff	IS; DS	IS: 86% 2 of which had malfunctioning recurring in only 4 days and required surgical replacement DS: 57%	Mild abdominal pain
Simons et al., 1999	Retrospective study	41 pt	Toronto Western, Tenckhoff	97% poor drainage; 30% poor inflow; 27% pain	Stiff guidewire and Amplatz extra-stiff wire in case of primary failure	Clinical success: functioning after 30 days	Clinical success: 55% Success of remanipulation: 63%	Peritonitis within 1 mo. after procedure (4 events)
Savader et al., 1999 [14]	Retrospective study	23 pt, 34 proc.	Tenckhoff catheters	30 cases inadequate drainage 4 cases painful dialysis	Angled Terumo/tip-deflecting wire/Bentson/angled stiff Terumo/Rosen 3 mm J/Amplatz 0.025 inch	Long-term catheter patency (>30 days); primary radiologic patency: time from first manipulation to a remanipulation/new CAPD catheter/switch of RRT/death; secondary radiologic patency: time from first manipulation to a remanipulation/new CAPD catheter/switch of RRT/death. Catheter patency after 3, 6, 12 mo	Long-term catheter patency: 58% Primary patency at 3,6 and 12 mo.: 0.61, 0.54, 0.11. Secondary patency at 3, 6, and 12 mo.: 0.75, 0.69, and 0.54, respectively	Post proc. peritonitis (1 pt)
Dobrashian et al., 1999 [15]	Retrospective study	18 pt, 23 proc.	Tenckhoff catheters	Catheter migration	Stainless steel wires made specifically for the procedure	Technical success (successful repositioning at screening) Clinical success (continued effective CAPD for at least 6 mo.s thereafter)	Technical success: 84%; clinical success 44%; 33% of the 15 pt whose manipulations were technically successful, the catheter remigrated within 6 mo., 4 of these were remanipulated, 1 pt required a third manipulation	Fragmentation of the tip of the balloon within the Tenckhoff catheter and peritoneal cavity (1 pt)
Lee et al., 2003 [8]	Retrospective study	22 pt	Straight Tenckhoff catheter	Catheter tip migration	Curved-tip, fixed-core guidewire with a polytetrafluoroethylene (PTFE) coating (Spring coil guidewire, 150 cm in length, with 3 mm J curve)	IS; DS	IS: 86%; DS: 59%	None
Ozyer et al., 2009 [9]	Retrospective study	12 pt, 14 proc.	Not reported	Migration to hypochondriac region	A hollow metal rod with three angles was made by reshaping the stiffener of a 14F drainage catheter (Flexima Regular APD All Purpose Drainage Catheter Set; Boston Scientific, Natick, MA, USA)	Technical success: reposition of the catheter tip DS: functional catheter remained within the true pelvis > 1 mo.	Technical success: 93% DS: 82% 2 remanipulations, both successful	Pain
Garcia-Mendez et al., 2012 [17]	Case series	3 pt	Not reported	Drainage difficulty	Not reported	None	2/3 guidewire relocated. 1/3 require surgical removal due to catheter entrapment	None
Saka et al., 2015 [12]	Retrospective study	23 pt	Double-cuffed, swan-neck catheters	22 outflow failure 1 outflow and inflow failure	Alpha replacer	Success: no relapse of outflow failure for at least 30 days after the proc.	60.8%	None
Asai et al., 2021 [13]	Retrospective study	8 pt.	Positioned with the Moncreid-Popovich implantation technique	5 outflow failure 2 outflow and inflow failure 1 omental wrapping	Alpha replacer	Restoration of flow	87.5%	None
Li et al., 2021 [6]	Retrospective study	35 pt	60 cm Quinton curled catheter	2 (5%) inflow obstruction 26 (63%) outflow obstruction 12 (29%) inflow and outflow obstruction	8.5-F biliary drainage catheter set (Cook Medical, Bloomington, Indiana); if necessary, two 145 cm, 0.035-inch hydrophilic guidewires (Terumo, Somerset, NJ, USA) with a 3 cm flexible tip	IS; clinical success: adequate function for at least 30 days after the procedure	IS: 87% for malpositioned catheters; 70% for obstructed; if both, 90%. Clinical success: 77% for malpositioned; 43% for obstructed; if both, 44% 56% of failed cases underwent surgical salvage	Exit site infection (1 pt); culture-negative peritonitis (1 pt)

IS: immediate success; DS: durable success; AKI: Acute Kidney Injury; PD: peritoneal dialysis; mo: month/months.; pt: patients; proc.: procedures; RRT: Renal Replacement Therapy; CAPD: Continuous Ambulatory Peritoneal Dialysis; PTFE: polytetrafluoroethylene.

## Data Availability

No new data were created.
